# Protamine sulphate for heparin reversal in percutaneous cardiac interventions: a systematic review and meta-analysis of randomized controlled trials

**DOI:** 10.1007/s00210-025-04369-4

**Published:** 2025-07-04

**Authors:** Elsayed Balbaa, Ahmed Farid Gadelmawla, Abdelrahman M. Tawfik, Ahmed Naeem, Ahmed Elbataa, Mohammad Bazzazeh, Ahmed Ramadan Fatiem, Khaled Ali, Obieda Altobaishat, Mohamed Abuelazm

**Affiliations:** 1 Faculty of Medicine, Alexandria University, Alexandria, Egypt; 2 Faculty of Medicine, Menoufia University, Menoufia, Egypt; 3 Faculty of Medicine, Al-Azhar University, Asyut, Egypt; 4 Faculty of Medicine, Al-Azhar University, Cairo, Egypt; 5 Internal Medicine, Rosalind Franklin University of Medicine and Science, Chicago, USA; 6 Faculty of Medicine, Jordan University of Science and Technology, Irbid, Jordan; 7 Faculty of Medicine, Tanta University, Tanta, Egypt

**Keywords:** Cardiac catheterization, Catheter ablation, Heparin, Hospital stay, PCI, TAVR

## Abstract

Vascular complications after percutaneous cardiac interventions are a significant cause of morbidity that affects post-procedural prognosis. Data about the efficacy and safety of routine protamine sulphate to prevent these complications remain scarce. Hence, we aim to assess the efficacy and safety of routine protamine sulphate use for heparin reversal in patients undergoing percutaneous cardiac interventions. We systematically searched PubMed, Embase, Cochrane, Scopus, and Web of Science for randomized controlled trials (RCTs) until September 2024. The RCTs’ quality was assessed using the Cochrane tool (RoB 2). Using R, we pooled dichotomous outcomes using risk ratios (RR) and continuous outcomes using mean differences (MD), both along the corresponding 95% Confidence interval (CI). PROSPERO ID: (CRD42024593002). Six RCTs with 1,076 patients were eligible. Hemostasis success was significantly higher in the protamine group (RR, 1.06; 95% CI [1.01–1.10]; *P* = 0.01), and the length of hospital stay was significantly shorter (MD, -0.46; 95% CI [-0.65, -0.26]; *P* < 0.01). There was no significant difference between both groups in stroke (RR, 0.60; 95% CI [0.18–2.03]; *P* = 0.41), major bleeding (*P* = 0.25), minor bleeding (*P* = 0.11), life-threatening bleeding (*P* = 0.65), time to ambulation (*P* = 0.13), all-cause mortality (*P* = 0.92), hematoma/pseudoaneurysm (*P* = 0.92), and major vascular complications (*P* = 0.70). While protamine improved hemostasis success, its effect on reducing bleeding events and time to ambulation was insignificant. Also, reducing the length of hospital stay in the protamine group highlights its potential clinical benefit.

**Graphical Abstract:**

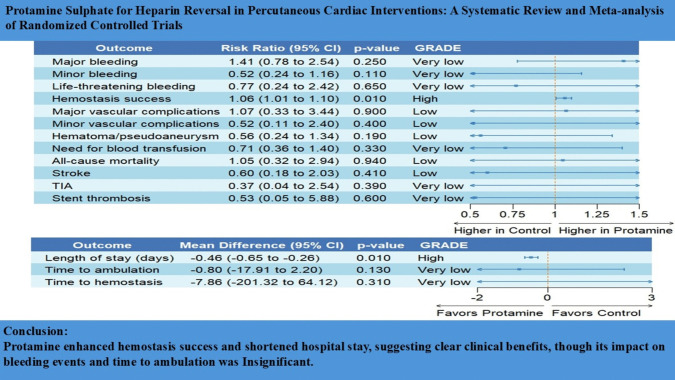

**Supplementary Information:**

The online version contains supplementary material available at 10.1007/s00210-025-04369-4.

## Introduction

There is a growing trend toward minimally invasive cardiac interventions (MICIs), including percutaneous coronary intervention (PCI), transcatheter aortic valve replacement (TAVR), and others (Balbaa 2025; Abdelazeem 2022). Approximately 36.8% and 55.7% of aortic and mitral valve surgeries now use MICI techniques (Ilcheva et al. 2023). Major bleeding remains one of the most critical risks associated with many MICIs. For example, some studies have reported a 6.2% bleeding risk with an associated mortality rate of 17.8% among patients with bleeding. In comparison, others have reported a haemorrhage-related mortality rate of up to 77% (Piccolo et al. 2021; Gerckens et al. 2017; Généreux et al. 2012).

Maintaining warfarin or novel oral anticoagulants before surgery and heparin use during surgery is the most widely used strategy to reduce thromboembolic risks and complications (Vriesendorp et al. 2024; Ghannam et al. 2018). However, at the end of the surgery, the metabolism of heparin required to achieve adequate vascular hemostasis can take a relatively long duration, thereby limiting patients’mobility, extending hospital stay, and increasing morbidity and costs of healthcare (Patel et al. 2022). To address this issue, protamine, a rapid-onset positively charged agent, can be administered to bind to the negatively charged heparin, forming an inert protamine-heparin complex that can easily be cleared from circulation. This rapid onset of action of protamine results in effective heparin reversal, allowing rapid hemostasis (Calkins et al. 2017; Sokolowska et al. 2016; Bromfield et al. 2013).

Protamine is regularly used in cardiac surgeries. However, there is limited evidence on its safety and efficacy for bleeding control in MICI. For example, some studies showed shorter hospital stays and either no or minor vascular complications with protamine use, while other studies found no significant reduction in major bleeding rates (Vriesendorp et al. 2024; Ghannam et al. 2018; Zbroński et al. 2021; Pan et al. 1997). This conflicting evidence highlights the need for further research to clarify the safety and efficacy of heparin reversal within the context of MICI. Therefore, we conducted this systematic review and meta-analysis to synthesize evidence from randomized controlled trials (RCTs) on the efficacy and safety of heparin reversal using protamine sulphate in patients undergoing MICIs.

## Methodology

### Protocol registration

Our review followed the Cochrane Handbook for Systematic Reviews and Meta-Analyses (Cochrane 2024) and was reported using the Preferred Reporting Items for Systematic Reviews and Meta-Analyses (PRISMA) guidelines (Page et al. 2021). This study’s protocol was published on PROSPERO and pre-registered (CRD42024593002).

### Data sources & search strategy

Through September 12th, 2024, two authors conducted an electronic search across five databases: PubMed (MEDLINE), Scopus, Web of Science (WoS), Cochrane Central Register of Controlled Trials (CENTRAL), and EMBASE. The search terms and results are presented in Table S1.

### Eligibility criteria

We included randomized controlled trials (RCTs) that followed the following PICO criteria: population (patients undergoing MICIs, including TAVR, PCI, catheter ablation, etc.); intervention: protamine sulphate, regardless of the dosing protocol; control (placebo or no protamine), and outcomes: the primary outcomes included major bleeding and hemostasis success (defined as hemostasis achieved within 20 min, without further need for manual compression), while the secondary outcomes included time to ambulation, time to hemostasis success, length of hospital stay, hematoma/pseudoaneurysm, minor bleeding, life-threatening bleeding, major vascular complications, minor vascular complications, need for blood transfusion, transient ischemic attack, stroke, and stent thrombosis, and all-cause mortality.

Studies meeting the following criteria were excluded: 1) non-original studies (e.g., book chapters, reviews, correspondence, letters to editors, commentaries, press articles, guidelines, etc.); 2) any other sort of study design except RCTs; 3) research with duplicate or overlapping datasets; 4) studies with a sample size of fewer than ten individuals; 6) in vitro experiments and non-human studies; and 7) studies not published in English.

### Study selection

The Covidence online tool was used to complete the review process. After removing duplicates, two authors (O.T. and A.T.) independently evaluated the retrieved records. During the full-text screening, two authors (A.F.G. and M.B.) assessed the entire text of the documents that initially met the eligibility criteria. Each disagreement was resolved through consensus and conversation with a senior author (M.A.).

### Data extraction

After retrieving all the relevant study texts, we performed a pilot extraction to ensure the data extraction sheet was properly organized. The data extraction sheet, formatted in Excel (Microsoft, USA), has two main sections. The summary characteristics of the included studies were reported in the first section (study ID, country, study design, blinding status, sample size, population, intervention, control, protamine dose, heparin dose, primary endpoints, and main findings). The second part included the baseline characteristics of the participants (age, sex, hypertension, diabetes, prior cardiac surgery, procedural time).

### Risk of bias and certainty of evidence

Two authors (A.R.F. and K.A.) independently assessed the study quality using the Cochrane RoB2 tool (Cochrane 2024). They evaluated the following five domains: the risk of bias in the randomization procedure, divergence from the intended intervention, missing outcome data, measuring the outcome, and selecting the reported outcomes. The senior author was consulted, and agreements were reached to resolve conflicts. We evaluated the degree of evidence’s certainty using the Grading of Recommendations Assessment, Development, and Evaluation (GRADE) guidelines(Guyatt 2009; Guyatt 2008). We considered publication bias, risk of bias, indirectness, inconsistency, and imprecision. Every result was evaluated, and the choices were supported by evidence and recorded.

### Statistical analysis

The study employed R version 4.3 for statistical analysis using the “meta,” and “metafor,” packages. With 95% confidence intervals, the analysis pooled data from multiple studies using either mean differences (for continuous outcomes) or risk ratios (for dichotomous outcomes). We used the chi-square test and quantified heterogeneity using the *I*-squared test, which quantifies the percentage of heterogeneity not attributed to chance. Heterogeneity was considered statistically significant if the chi-square test p-value was less than 0.1. When chi-square and *I*-square tests revealed significant heterogeneity (I2 > 50%), a random-effects model (DerSimonian-Laird (DL) method) was employed; otherwise, a common-effect model was used. An *I*^2^value of 0–40% indicated low heterogeneity, 30–60% showed moderate heterogeneity, 50–90% probably represented high heterogeneity, and 75–100% suggested very high heterogeneity, following the Cochrane Handbook (Cumpston 2019). In case of substantial heterogeneity, whenever feasible, we did a sensitivity analysis, omitting one study at a time, and reran the analysis to investigate the source of heterogeneity. We also did a sensitivity analysis using the leave-one-out method whenever possible to test the robustness of the results statistically. We conducted a subgroup analysis according to procedure type whenever possible. Finally, the test for publication bias was not feasible as we included fewer than ten studies in all comparisons (Egger 1997).

## Results

### Search results and study selection

**Fig. 1:**
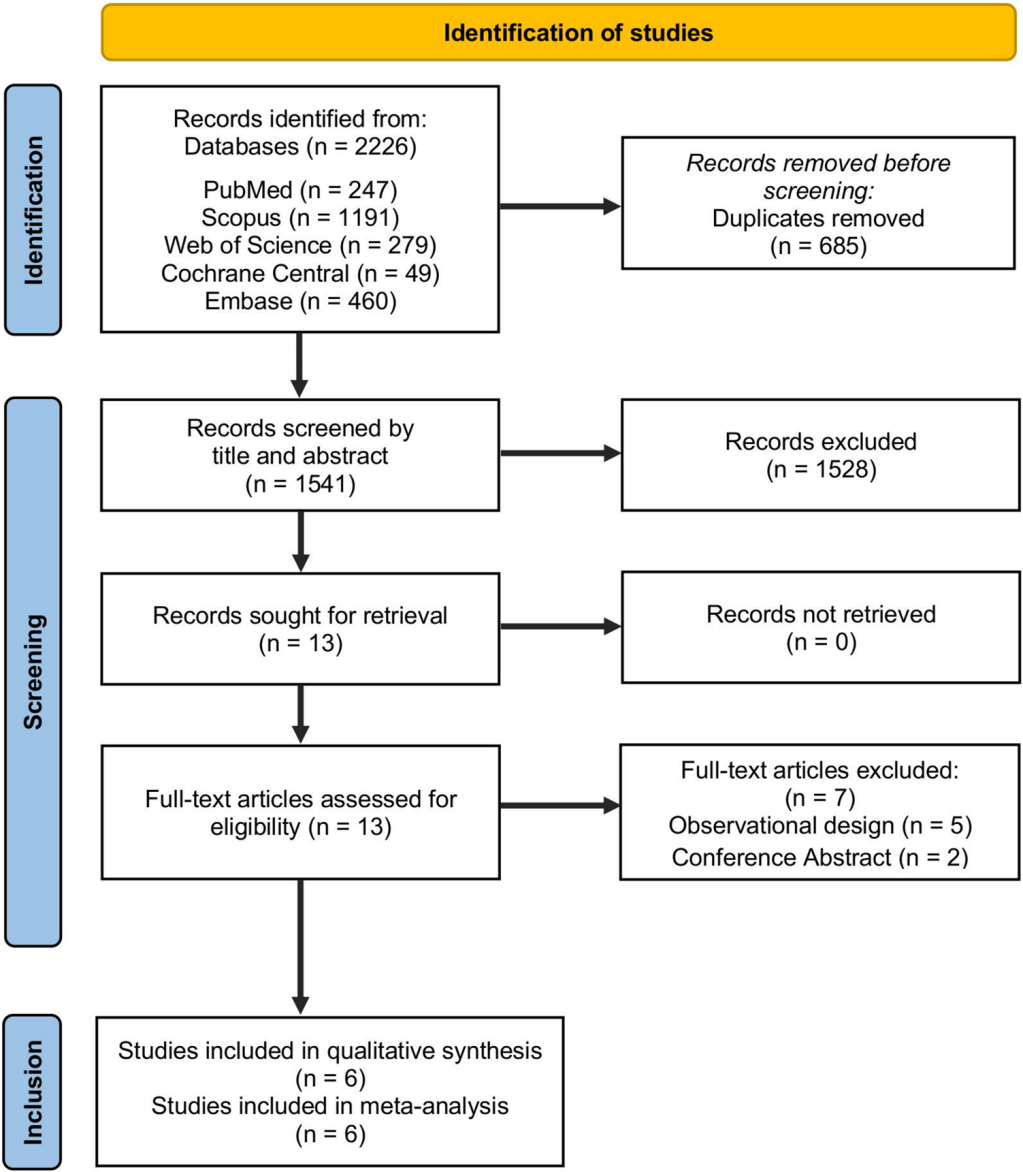
PRISMA flow chart of the screening process

Our comprehensive literature search retrieved 2,226 articles, and 685 references were excluded after duplicate removal, leaving 1,541 references for primary screening by title and abstract. After primary screening, 24 articles were available to be assessed by full-text reviews for our eligibility criteria. Finally, we included six RCTs (Pan 1997; Lohne et al. 2004;Kaneda et al. 2005; Ghannam et al. 2018; Zbroński et al. 2021; Vriesendorp et al. 2024) with 1,076 patients in this systematic review and meta-analysis. The PRISMA flowchart of the selection process is shown in Fig. 1.

### Characteristics of included studies and baseline data

Two of the included studies enrolled patients undergoing TAVI(Zbronki 2021; Vriesendorp 2024), another two enrolled those undergoing PCI with stent implantation (Pan 1997; Kaneda 2005), one enrolled those undergoing PCI angioplasty (Lohne 2004), and another enrolled those undergoing catheter ablation for atrial fibrillation (Ghannam et al. 2018). Sheaths were introduced through transfemoral access in all the included studies. More details about the characteristics of the included studies and the patients’baseline data are provided in Tables1 and 2.

**Table 1:** Summary of the included studies

Study ID	Country (centers)	Design	Sample Size	Type of procedure	Intervention	Control	Access	Protamine Dose (mean ± SD or value)	Heparin Dose, UI (mean ± SD or value)	Primary Endpoints	Main Findings
Vriesendorp et al. 2024	Australia (3 centers)	RCT	Total: 410 Protamine: 199 Control: 211	TAVR	Protamine sulfate	Placebo	Transfemoral	100 mg	9000 ± 1493	Rate of homeostasis success and time to homeostasis	Protamine significantly improved the success rate of homeostasis and reduced the time to homeostasis, minor vascular complications, procedural time, and hospital stay compared to placebo patients
Zbronski et al. 2021	Poland (1 center)	RCT	Total: 100 Protamine: 47 Control: 53	TAVR	Protamine sulfate	Placebo	Transfemoral	36.67 ± 19.12 mg	7000 ± 1530	life-threatening and major bleeding complications	Protamine sulfate did not significantly reduce major and life-threatening bleeding complications post-TAVI
Ghannam et al. 2018	USA (1 center)	RCT	Total: 150 Protamine: 77 Control: 73	Catheter Ablation for AF	Protamine sulfate	No protamine	Transfemoral	48.8 ± 11.1 mg	23,700 ± 13,977	Ambulation after the termination of the procedure	Protamine can expedite vascular hemostasis and ambulation time after CA of AF or atrial flutter without increasing complications and should be routinely considered after CA
Kaneda et al. 2005	Japan (1 center)	RCT	Total: 88 Protamine: 43 Control: 45	PCI – Stent implantation	Protamine	No protamine	Transfemoral	100 mg	10,000	Major bleeding, minor bleeding, subacute stent thrombosis	Protamine is safe and feasible to reduce bleeding complications in stable angina patients receiving aspirin and ticlopidine antiplatelet regimens
Lohne et al 2004	Norway (1 center)	RCT	Total: 100 Protamine: 50 Control: 50	PCI – Angioplasty	Protamine + immediate sheath removal	No protamine + delayed sheath removal	Transfemoral	24 ± 6 mg (10 or 20 or 30)	10,000	Time to mobilization, patient comfort, puncture site hematoma, coronary events	Protamine improved patient comfort and reduced immobilization time, but cardiac safety concerns necessitate the administration of antiplatelet agent clopidogrel before the procedure
Pan et al. 1997	Spain (2 centers)	RCT	Total: 228 Protamine: 117 Control: 111	PCI – Stent implantation	Protamine + immediate sheath removal	No protamine + delayed sheath removal	Transfemoral	2 mg/kg	10,000	Outside laboratory vessel occlusion, death, stent thrombosis, systemic bleeding, groin hematoma or pseudoaneurysm, and hospital stay	Protamine can be safely administered after stent implantation to neutralize circulating heparin, benefiting patients with hemorrhagic risk and reducing hospital stay time

Abbreviations**:** *AF*, atrial fibrillation; *CA*, catheter ablation; *NR*, not reported; *PCI*, percutaneous coronary intervention; *RCT*, randomized controlled trial; *SD*, standard deviation; *TAVR*, transcatheter aortic valve replacement

**Table 2:** Baseline data of the included patients

Study ID	Group	Age (years), (Mean ± SD)	Sex – Male (%)	Hypertension (%)	Diabetes (%)	Prior Cardiac Surgery (%)	Procedural time (Mean ± SD)	VKA use (%)	DOAC use (%)	Peak ACT Time (Mean ± SD)	ACT time at sheath removal (Mean ± SD)
Vriesendorp et al. 2024	Protamine	81.30 ± 5.98	66	78	33	18	59 ± 15.6	0	10.6	286 ± 41.67	204.3 ± 214.3
	Control	80 ± 7.47	58	82	23	16	61.6 ± 15.6	0	5.7	287 ± 32.84	283.3 ± 34.3
Zbronski et al. 2021	Protamine	81.27 ± 6.12	47	87	51	15	NR	NR	NR	NR	NR
	Control	80.67 ± 8.38	49	87	36	9	NR	NR	NR	NR	NR
Ghannam et al. 2018	Protamine	63 ± 12	60	NR	NR	NR	199 ± 74	18	82	359 ± 31	152 ± 25
	Control	66 ± 9	66	NR	NR	NR	214 ± 140	19	81	359 ± 29	176 ± 13
Kaneda et al. 2005	Protamine	65 ± 10	84	23	12	NR	NR	NR	NR	NR	NR
	Control	66 ± 10	76	11	11	NR	NR	NR	NR	NR	NR
Lohne et al 2004	Protamine	65 ± 11	62	NR	NR	NR	NR	NR	NR	NR	NR
	Control	63 ± 10	70	NR	NR	NR	NR	NR	NR	NR	NR
Pan et al. 1997	Protamine	59 ± 9	82	52	19	4	NR	NR	NR	NR	NR
	Control	59 ± 10	84	47	26	2	NR	NR	NR	NR	NR

Abbreviations: *ACT*, activated clotting time; *DOAC*, direct oral anticoagulants; *NR*, not reported; *SD*, standard deviation; *VKA*, Vitamin K antagonist

### Risk of bias and certainty of evidence

**Fig. 2:**
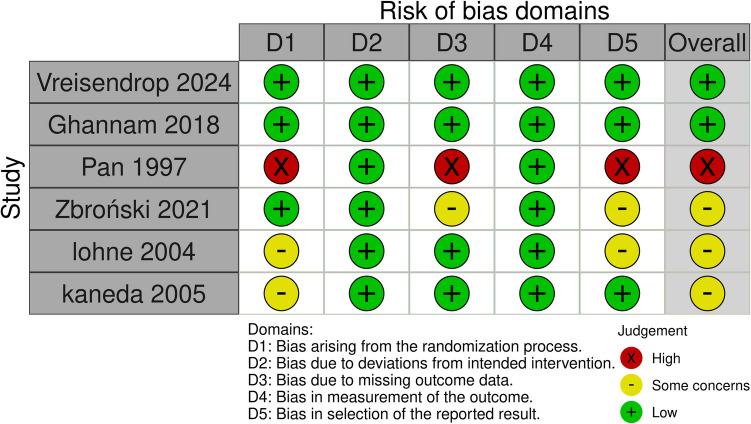
Quality assessment of risk of bias in the included trials. This panel presents a schematic representation of risks (low = green, unclear = yellow, and high = red) for specific types of biases of each of the studies in the review

Overall, two studies showed a low risk of bias (Vriesendorp et al. 2024 and Ghannam et al. 2018); three studies showed some concerns of bias (Kaneda et al. 2005; Lohne et al. 2004; Zbroński et al. 2021); and another study showed a high risk of bias (Pan et al. 1997). Regarding Pan et al. (Pan 1997) There was no clear information about randomization, allocation concealment, or blinding. In Kaneda et al. (2005) and Lohne et al. (2004). There was no clear information about the randomization process. There was selective outcome reporting in Lohne et al. (2004) and Zbroński et al. (2021) (Ghannam et al. 2018). Also, the outcome assessors were not blinded in Zbroński et al. (2021) (Fig. 2). Table 3 summarizes the certainty of evidence assessed according to the GRADE guidelines.

**Table 3:** GRADE evidence profile

Certainty assessment							Summary of findings				
Participants (studies)	Risk of bias	Inconsistency	Indirectness	Imprecision	Publication bias	Overall certainty of evidence	Study event rates (%)		Relative effect (95% CI)	Anticipated absolute effects (per 1000)	
							With [Protamine]	With [Control]		Risk with Protamine	Risk difference with Control (95% CI
Major Bleeding											
811 (4 RCTs)	serious^a^	not serious	not serious	very serious^b,c^	None	⨁◯◯◯ Very low	18/399 (4.5%)	14/412 (3.4%)	RR 1.41 (0.78 to 2.54)	48	14 more (7 lower to 52 more)
Hemostasis success											
469 (2 RCTs)	not serious	not serious	not serious	not serious	None	⨁⨁⨁⨁ High	231/239 (96.65%)	234/256 (91.4%)	RR 1.06 (1.01 to 1.10)	969	55 more (from 9 to 91 more)
Major vascular complications											
645 (3 RCTs)	not serious	not serious	not serious	very serious^b,c^	None	⨁⨁◯◯ Low	5/316 (1.6%)	5/329 (1.5%)	RR 1.07 (0.33 to 3.44)	16	1 more (from 10 lower to 37 more)
Minor vascular complications											
545 (2 RCTs)	not serious	serious^d^	not serious	very serious^b,c^	None	⨁⨁◯◯ Low	9/269 (3.3%)	21/276 (7.6%)	RR 0.52 (0.11 to 2.40)	40	36 lower (from 68 lower to 106 more)
Minor bleeding											
583 (3 RCTs)	serious^a^	not serious	not serious	very serious^b,c^	None	⨁◯◯◯ Very low	8/282 (2.8%)	17/301(5.6%)	RR 0.52 (0.24 to 1.16)	29	27 lower (from 43 lower to 9 more)
Life-threatening bleeding											
328 (2 RCTs)	serious^a^	not serious	not serious	very serious^b,c^	None	⨁◯◯◯ Very low	4/164 (2.4%)	6/164 (3.7%)	RR 0.77 (0.24 to 2.42)	28	9 lower (from 28 lower to 53 more)
Hematoma and pseudoaneurysm formation											
873 (4 RCTs)	not serious	not serious	not serious	very serious^b,c^	None	⨁⨁◯◯ Low	8/436 (1.8%)	14/437 (3.2%)	RR 0.56 (0.24 to 1.34)	18	14 lower (from 24 lower to 11 more
All-cause mortality											
973 (5 RCTs)	not serious	not serious	not serious	very serious^b,c^	None	⨁⨁◯◯ Low	6/483 (1.2%)	6/490 (1.2%)	RR 1.05 (0.38 to 2.94)	13	1 more (from 7 lower to 23 more)
Need for blood transfusion											
200 (2 RCTs)	serious^a^	not serious	not serious	very serious^b,c^	None	⨁◯◯◯ Very low	10/97 (10%)	16/103 (16%)	RR 0.71 (0.36 to 1.40)	114	46 lower (from 102 lower to 64 more)
TIA											
518 (2 RCTs)	serious^a^	not serious	not serious	very serious^b,c^	None	⨁◯◯◯ Very low	1/246 (0.04%)	3/272 (1%)	RR 0.37 (0.04 to 3.54)	4	6 lower (from 9 lower to 35 more)
Stroke											
660 (3 RCTs)	not serious	not serious	not serious	very serious^b,c^	None	⨁⨁◯◯ Low	3/323 (0.9%)	6/273 (2.1%)	RR 0.60 (0.18 to 2.03)	13	8 lower (from 4 lower to 22 more)
Stent thrombosis											
328 (2 RCTs)	serious^a^	not serious	not serious	very serious^b,c^	None	⨁◯◯◯ Very low	1/164 (0.6%)	2/164 (1.2%)	RR 0.53 (0.05 to 5.88)	6	6 lower (from 11 lower to 59 more)
Length of hospital stay											
744 (3 RCTs)	not serious	not serious	not serious	not serious	None	⨁⨁⨁⨁ High	363	381	-	-	MD 0.46 lower (from 0.65 lower to 0.26 lower
Time to hemostasis success											
572 (2 RCTs)	not serious	very serious^e^	not serious	serious^b^	None	⨁◯◯◯ Very low	269	276	-	-	MD 68.6 lower (from 201.32 lower to 64.12 higher)
Time to ambulation											
250 (2 RCTs)	not serious	very serious^e^	not serious	serious^b^	None	⨁◯◯◯ Very low	127	123	-	-	MD 7.68 lower (from 17.91 lower to 2.20 higher)

Abbreviations: *CI*, Confidence interval; *MD*, Mean difference; *RR*, Risk ratio; *RCT*, Randomized clinical trial

^a^High risk or unclear bias

^b^A wide confidence interval that does not exclude the appreciable harm/benefit

^c^Low number of events (< 300 events)

^d^I2 > 50%

^e^I2 > 90%

### Primary outcomes

#### Major bleeding

**Fig. 3:**
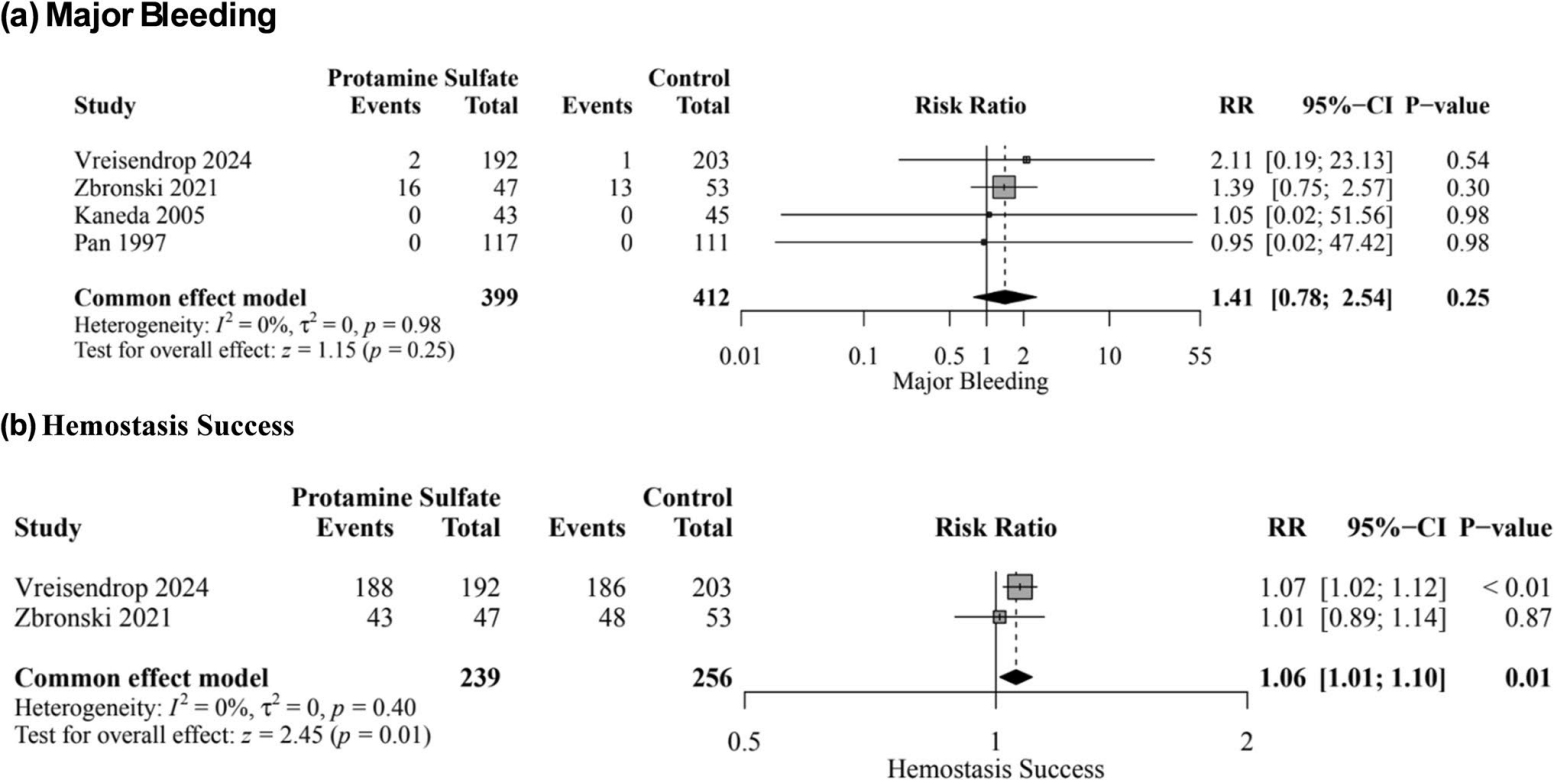
Forest plot of the primary outcomes; major bleeding (**a**), and hemostasis success (**b**); RR: risk ratio, CI: confidence interval

Four studies, enrolling 811 patients, reported major bleeding events. There was no significant difference in major bleeding risk between protamine sulfate and the control groups (RR, 1.41; 95% CI [0.78–2.54]; *P* = 0.25; Fig. 3a), with no heterogeneity observed among the included studies (*I*^2^ = 0%, *P* = 0.98). The pooled results were robust through sensitivity analysis.

#### Hemostasis success

Two studies enrolling 495 patients reported hemostasis success. The protamine sulfate group was significantly associated with an increase in hemostasis success rate compared to the control group (RR, 1.06; 95% CI [1.01–1.10]; *P* = 0.01; Fig. 3b), with no heterogeneity observed among the included studies (*I*^2^ = 0%, *P* = 0.40).

### Secondary outcomes

**Fig. 4:**
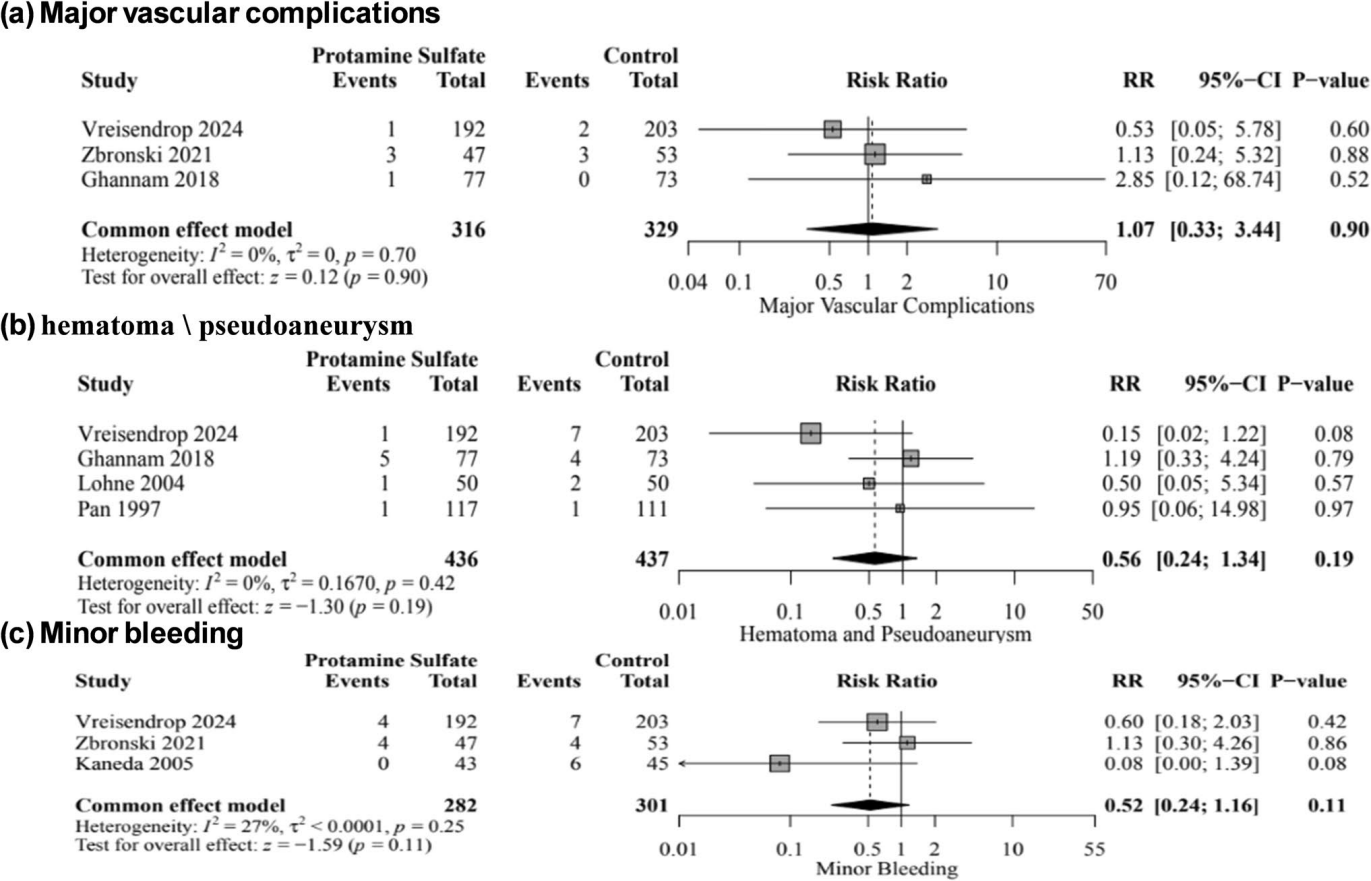
Forest plot of Major vascular complications (**a**), Hematoma and pseudoaneurysm formation (**b**), and minor bleeding (**c**); RR: risk ratio, CI: confidence interval

**Fig. 5:**
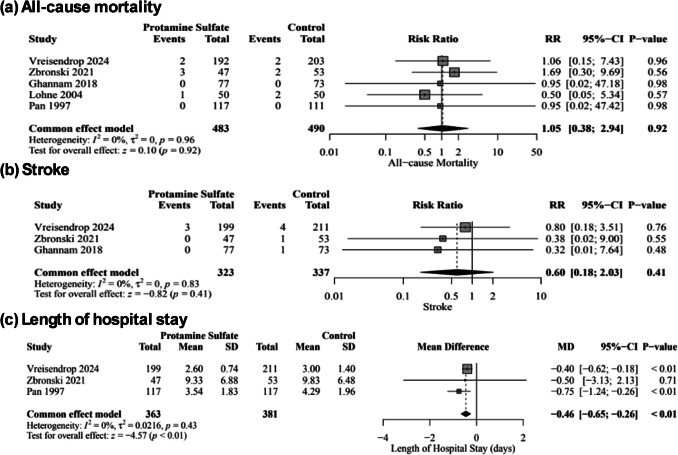
Forest plot of the All-cause mortality (**a**), stroke (**b**), and length of hospital stay (**c**); RR: risk ratio, MD: mean difference, SD: standard deviation, CI: confidence interval

There was no significant difference between protamine sulfate and the control groups in major vascular complications risk (RR, 1.07; 95% CI [0.33–3.44]; *P* = 0.90; Fig. 4a), hematoma and pseudoaneurysm formation (RR, 0.56; 95% CI [0.24–1.34]; *P* = 0.19; Fig. 4b), minor bleeding (RR, 0.52; 95% CI [0.24–1.16]; *P* = 0.11; Fig. 4c), life-threatening bleeding (RR, 0.77; 95% CI [0.24–2.42]; *P* = 0.65; Fig. S1), minor vascular complications (RR, 0.52; 95% CI [0.11–2.40]; *P* = 0.40; Fig. S2), time to hemostasis success (MD, −68.60; 95% CI [−201.32, 64.12]; *P* = 0.31; Fig. S3), time to ambulation (MD,; 95% CI [, 2.20]; *P* = 0.13; Fig. S4), all-cause mortality (RR, 1.05; 95% CI [0.38–2.94]; *P* = 0.92; Fig. 5a), need for blood transfusion (RR, 0.71; 95% CI [0.36–1.40]; *P* = 0.33; Fig. S5), TIA (RR, 0.37; 95% CI [0.04–3.54]; *P* = 0.39; Fig. S6), stroke (RR, 0.60; 95% CI [0.18–2.03]; *P* = 0.41; Fig. 5b), and stent thrombosis (RR, 0.53; 95% CI [0.05–5.88]; *P* = 0.60; Fig. S7). However, protamine sulfate was associated with a significant decrease in the length of hospital stay compared to the control group (MD, −0.46; 95% CI [−0.65, −0.26]; *P* < 0.01; Fig. 5c). Studies were homogenous (*I*^2^ = 0%) in major vascular complications, hematoma and pseudoaneurysm formation, all-cause mortality, life-threatening bleeding, Stroke, transient ischemic attack, Stent thrombosis, need for blood transfusion, and length of hospital stay. On the other hand, there was low heterogeneity (*I*^2^ = 27%) in minor bleeding, high heterogeneity (*I*^2^ = 70%) in minor vascular complications, and very high heterogeneity and very high heterogeneity in time to hemostasis success (*I*^**2**^ = 99%) and time to ambulation (*I*^**2**^ = 100%). Sensitivity analysis by the leave-one-out method showed the robustness of all results, as no omission of a single study affected the significance (Figs. S8, S9, S10, S11, S12 and S13).

### Subgroup analysis based on MICI type

The test for subgroup analysis according to procedure type revealed no significant differences between subgroups in terms of major bleeding (*P* = 0.79, Fig. S14), all-cause mortality (*P* = 0.79, Fig. S15), major vascular complications (*P* = 0.67, Fig. S16), hematoma/pseudoaneurysm formation (*P* = 0.25, Fig. S17), minor bleeding (*P* = 0.13, Fig. S18), stroke (*P* = 0.65, Fig. S19), and the length of hospital stay (*P* = 0.19, Fig. S20).

### Hemodynamic and immunologic adverse effects of protamine

Only two of the included studies provided enough details about adverse events: Ghannam et al. (2018) reported one case (1.3%) of hypotensive response following protamine test dosage, after which the therapeutic dose was interrupted. Pan et al. (1997) reported skin rash in two patients (2%) and hypotension in six patients (5%) as well. A pooled analysis was not feasible.

## Discussion

This systematic review and meta-analysis study provides comprehensive evidence regarding using protamine sulphate to reverse heparin action in patients undergoing percutaneous cardiac catheterization. The efficacy and safety of protamine sulphate for reversing heparin action have been established in cardiac surgery (Puis et al. 2020; Boer 2018). However, protamine use is still controversial in interventional cardiology (Yamamoto et al. 2018; Kubota et al. 2020). Based on 1,076 patients undergoing MICIs obtained from six RCTs, the pooled analyses showed a significantly higher hemostasis success rate in the protamine sulphate group and a significant decrease in the length of hospital stay. Moreover, using protamine sulphate showed no significant differences when compared to the control group in the risks of major bleeding, minor bleeding, time to ambulation, time to hemostasis success, need for blood transfusion, all-cause mortality, hematoma formation, stroke, and stent thrombosis, all of which could highlight the safety associated with its use.

In this study, using protamine sulphate to reverse heparin action was significantly associated with a marginal increase in the hemostasis success rate compared to control, based on two studies that utilized TAVR. Heparin is used in cardiac catheterization to prevent thrombus formation through its anticoagulant effects (Wittkowsky 2002). Protamine sulphate effectively reverses the anticoagulant properties of heparin by neutralizing its negative charges through the formation of a stable heparin-protamine complex that competes with heparin on antithrombin III, platelet factor 4, and others, hence antagonizing its action (Maurer et al. 2011;Sokolowska et al. 2016). This is important in cardiac catheterization patients to facilitate quicker clot formation and achieve hemostasis (Capodanno 2022).

Moreover, a proper protamine-to-heparin dosing ratio is vital for achieving better hemostatic outcomes (Ho 2021)—a previous study by Meesters et al. compared high and low protamine-to-heparin dosing ratios in cardiac surgeries. A protamine-to-heparin dosing ratio of 1.3:1.0, which was regarded as a high dosing ratio, was associated with worse hemostasis outcomes; hence, differences in protamine-to-heparin dosing ratios across studies could be translated into differences in hemostasis success rates (Meesters et al. 2016) Notably, we found no significant differences in the time needed to achieve hemostasis success when comparing protamine sulphate to control groups. This means that while protamine sulphate is associated with a higher hemostasis success rate, it does not necessarily accelerate the process. However, this finding should be interpreted with caution because of the observed heterogeneity for this outcome.

As pooled from four studies, no significant differences were found in major bleeding risks between the protamine sulphate and control groups. We performed a sensitivity analysis and found no significant differences in the risk of major bleeding. This finding contradicts that of the previous two meta-analyses by De Luca et al. and Lee et al. which investigated the safety and efficacy of routine protamine administration for reversing heparin action in MICIs based on observational studies and RCTs (Lee et al. 2023; De Luca et al. 2010). In both studies, protamine significantly decreased the risk of major bleeding complications. However, Lee et al. highlighted the presence of moderate heterogeneity in this outcome (Lee et al. 2023).

A plausible explanation for this contradiction is using randomization techniques to classify MICIs patients into protamine and control groups in all four studies included in our analysis. This is not the case in meta-analyses by De Luca et al. and Lee et al. This potentially negates the role of selection bias and confounding bias that could give false estimates (Stukel 2007). The findings of Al-Kassou et al. could further augment this explanation; they conducted an observational study on patients undergoing TAVI, and protamine sulphate showed significantly lower rates of major bleeding complications, whereas the allocation of patients into protamine sulphate and control groups was left to operators’ decisions (Al-Kassou et al. 2020). Another possible explanation is the differences in protamine dosage across different studies. Excessive protamine could result in paradoxical anticoagulation through a multifactorial mechanism, leading to abnormal platelet function, stimulation of clot dissolution, and interference with different coagulation factors, leading to an increased risk of bleeding (Crivellari 2024; Mochizuki 1998). This explanation is augmented by the fact that using tailored protamine doses according to each patient’s requirements was associated with less bleeding and better coagulation (Koster et al. 2014; Vonk et al. 2014). Though protamine did not show an increased risk for major, minor, and life-threatening bleeding, previous research highlighted other possible adverse effects that should be kept into consideration, like bradycardia, hypotension, anaphylaxis, and pulmonary hypertension (Sokolowska et al. 2016). These adverse effects are associated with mild hemodynamic instability and could lead to fatal cardiovascular collapse and increased mortality risk (Welsby 2005; Nybo et al. 2008).

Our analysis showed no significant differences between the two groups in the risk of developing hematoma and stroke, as pooled from four and three studies, respectively, with no observed heterogeneity. These findings partially contradict those of Kakisis et al. (2016) who found protamine sulphate to be associated with a significant decrease in the risk of wound hematoma without increasing the risk of postoperative stroke for patients undergoing carotid endarterectomy (Nybo et al. 2008).This could be explained by differences in the dosage and timing of protamine administration in these two clinical situations and differences in the underlying pathophysiology of the two conditions.

Our analysis showed a significant decrease in the length of hospital stay for the protamine sulphate group compared to the control group. The lack of heterogeneity and robustness of sensitivity analysis further augments the role of heparin reversal in reducing hospitalization. This could be explained by the rapid arterial sheath removal on protamine administration and earlier patients’ discharge (Hanna et al. 2011). Early discharge benefits the patient and the healthcare system, as it is associated with a reduced risk of hospital-acquired infections and reduced hospitalization costs (Restelli 2019).

Notably, we found no significant difference in the ambulation time between the protamine and control groups, highlighting that although it accelerates hospital discharge, it has nothing to do with postoperative recovery. We also found no significant differences in stent thrombosis between the two groups. This finding is identical to that of Kubota et al. in which patients receiving protamine for heparin reversal had no increased risk for stent thrombosis (Kubota et al. 2020). This is also like that of Ducas et al. where early protamine administration was associated with early safe catheter removal and fewer local complications (Ducas et al. 2002). This may be due to the smaller stent sizes and the lower need for heparin reversal associated with these minimally invasive techniques (M ten 2020).

### Strengths

This study provided up-to-date evidence regarding protamine sulphate for heparin reversal in MICIs, representing the most comprehensive evidence concerning multiple outcomes. This meta-analysis was based on RCTs to ensure high-quality evidence while following the Cochrane Handbook for Systematic Reviews and Meta-Analyses and PRISMA guidelines. Additionally, we performed sensitivity analyses, ensuring that the pooled estimates were not significantly dependent on a single study, and heterogeneity analyses to quantify the variability across the studies.

### Limitations

Although this study provides comprehensive insights into the efficacy and safety of protamine sulphate for reversing heparin action in MICIs based on RCTs, few limitations are evident. This study was based on only six RCTs with multiple outcomes pooled from fewer studies. In addition, heterogeneity was observed across some outcomes, including microvascular complications and ambulation time. Moreover, slight variability in the patient population, procedural approaches, and protamine-to-heparin dosing ratio could have affected the pooled estimates. Lastly, the included studies covered a large time window (1997–2024), possibly including outdated techniques and practices, and making newer studies benefit more from recent advances in Cardiac Catheterization.

### Clinical implications

Protamine sulphate effectively enhances hemostasis in cardiac catheterization patients by reversing heparin action. Protamine sulphate use is associated with decreased hospitalization, which could reduce the risk of acquiring infections and reduce hospitalization-associated costs. The administration of protamine did not increase the risk of major bleeding, which is reassuring for clinicians considering protamine for heparin reversal. In addition, no significant differences were observed in hematoma, stroke, and stent thrombosis, suggesting that protamine could be administered safely without raising concerns about these adverse events. Clinicians should consider routine protamine administration, especially in high-risk cases, to ensure safety, reduce complications, and achieve better perioperative management while paying attention to the protamine-to-heparin ratio to minimize the risk of paradoxical anticoagulation. Based on the observed significant difference in hemostasis success and the absence of significant differences in bleeding outcomes, clinicians should consider other factors for bleeding, such as underlying bleeding tendencies, procedural techniques, and vascular access-associated bleeding.

## Conclusion

In patients undergoing MICIs, protamine sulphate was associated with higher hemostasis success rates and reduced hospitalization time. While the overall impact on bleeding and recovery time may be limited, protamine sulphate use did not pose an increased risk of stent thrombosis, stroke, transient ischemic attacks, or hematoma formation, all of which highlight its safety in the setting of cardiac catheterization. Owing to the limited number of pooled studies for some outcomes, more RCTs are needed to provide a more comprehensive understanding of the safety of protamine sulphate.

## Supplementary Information

Below is the link to the electronic supplementary material.

ESM 1 (DOCX 2.23 MB)

## Data Availability

All source data for this work (or generated in this study) are available upon reasonable request.
