# Supplementary material for: Protamine sulphate for heparin reversal in percutaneous cardiac interventions: a systematic review and meta-analysis of randomized controlled trials
Source: Naunyn Schmiedebergs Arch Pharmacol. 2025 Jul 4;398(12):16507–20. doi: 10.1007/s00210-025-04369-4 (PMC12678601; doi:10.1007/s00210-025-04369-4)

**Supplementary material.**

**Title.**

Protamine Sulfate for Heparin Reversal in Percutaneous Cardiovascular Interventions: A Systematic Review and Meta-analysis of Randomized Controlled Trials

**Authors.**

Elsayed Balbaa^1^, Ahmed Farid Gadelmawla^2^, Abdelrahman M. Tawfik^1^, Ahmed Naeem^3^, Ahmed Elbataa^3^, Mohammad Bazzazeh^1^, Ahmed Ramadan Fatiem^1^, Khaled Ali^4^, Obieda Altobaishat^5^, Mohamed Abuelazm^6^

**Affiliations.**

1. Faculty of Medicine, Alexandria University, Alexandria, Egypt
2. Faculty of Medicine, Menoufia University, Menoufia, Egypt
3. Faculty of Medicine, Al-Azhar University, Cairo, Egypt
4. Internal Medicine, Rosalind Franklin University of Medicine and Science, Chicago, USA
5. Faculty of Medicine, Jordan University of Science and Technology, Irbid, Jordan
6. Faculty of Medicine, Tanta University, Tanta, Egypt

**Corresponding author.**

Elsayed Balbaa

Email: [elsayedbalbaa@gmail.com](mailto:elsayedbalbaa@gmail.com)

**Table S1**: Search strategy.

| Database | Search Terms | Search Field | Search Results |
| --- | --- | --- | --- |
| PubMed | (("Protamine" OR "heparin reversal") AND ("Percutaneous" OR "transcatheter" OR "transcutaneous" OR "endovascular" OR "Transluminal" OR "angioplasty" OR"stent" OR "PCI" OR "TAVI" OR "TAVR" OR "atherectomy" OR "balloon dilation" OR "ablation")) | All Field | 247 |
| Cochrane | (("Protamine" OR "heparin reversal") AND ("Percutaneous" OR "transcatheter" OR "transcutaneous" OR "endovascular" OR "Transluminal" OR "angioplasty" OR"stent" OR "PCI" OR "TAVI" OR "TAVR" OR "atherectomy" OR "balloon dilation" OR "ablation")) | All Field | 49 |
| WOS | (("Protamine" OR "heparin reversal") AND ("Percutaneous" OR "transcatheter" OR "transcutaneous" OR "endovascular" OR "Transluminal" OR "angioplasty" OR"stent" OR "PCI" OR "TAVI" OR "TAVR" OR "atherectomy" OR "balloon dilation" OR "ablation")) | All Field | 279 |
| SCOPUS | (("Protamine" OR "heparin reversal") AND ("Percutaneous" OR "transcatheter" OR "transcutaneous" OR "endovascular" OR "Transluminal" OR "angioplasty" OR"stent" OR "PCI" OR "TAVI" OR "TAVR" OR "atherectomy" OR "balloon dilation" OR "ablation")) | Title, Abstract, Keywords | 1191 |
| EMBASE | (("Protamine" OR "heparin reversal") AND ("Percutaneous" OR "transcatheter" OR "transcutaneous" OR "endovascular" OR "Transluminal" OR "angioplasty" OR"stent" OR "PCI" OR "TAVI" OR "TAVR" OR "atherectomy" OR "balloon dilation" OR "ablation")) | All Field | 460 |

**Figure S1:** Life-threatening bleeding.

**
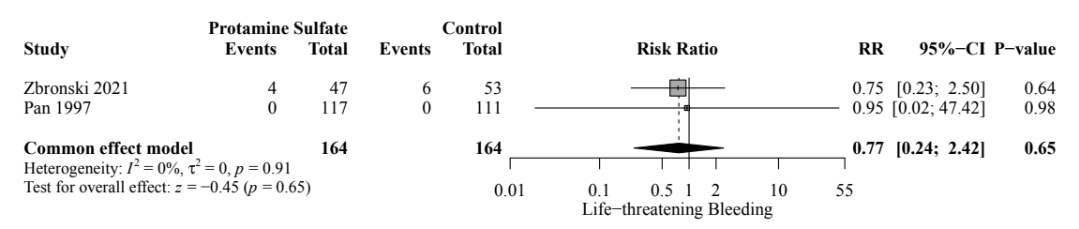
**

**Figure S2:** Minor vascular complications.

**
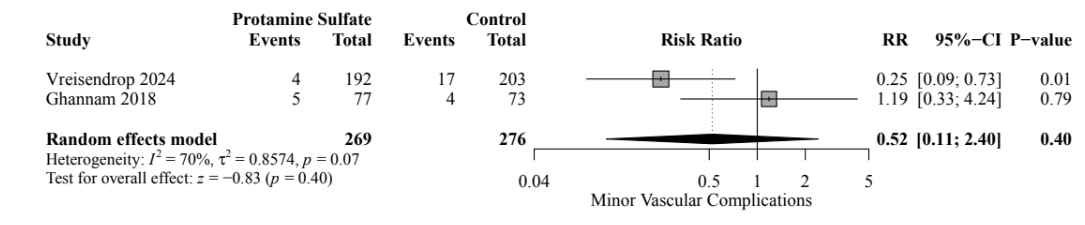
**

**
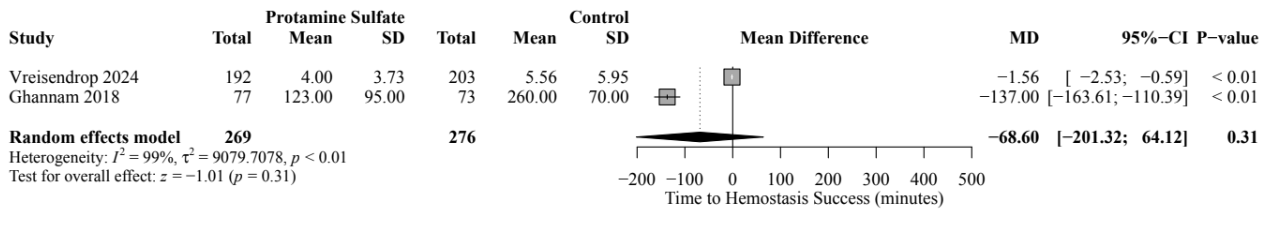
Figure S3:** Time to hemostasis success.

**Figure S4:** Time to ambulation.

**
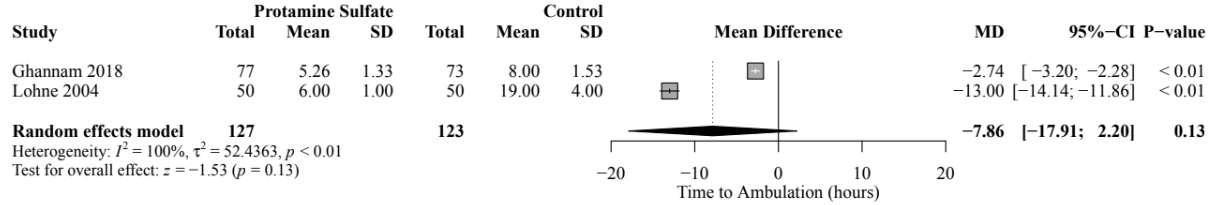
**

**Figure S5:** Need for blood transfusion.

**
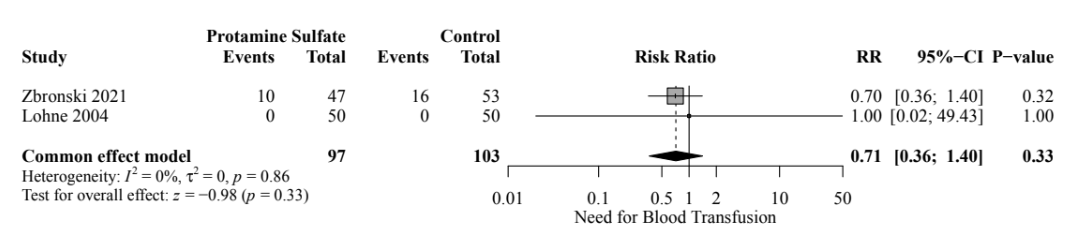
**

**Figure S6:** Transient ischemic attack (TIA).

**
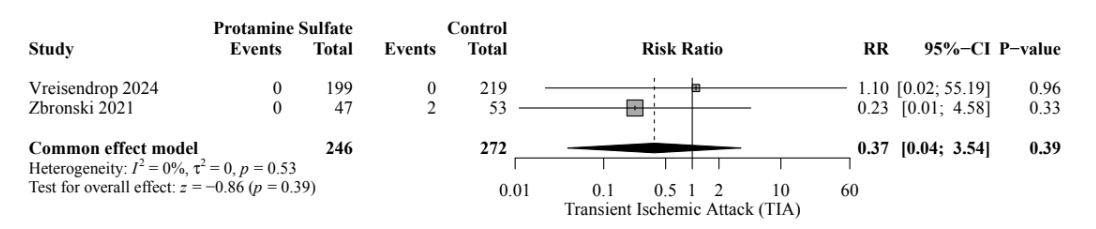
**

**Figure S7:** Stent thrombosis.

**
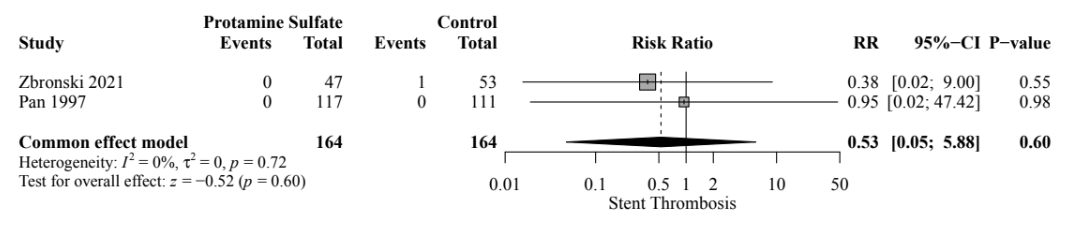
**

**Figure S8:** Leave-one-out sensitivity analysis of Major Bleeding.


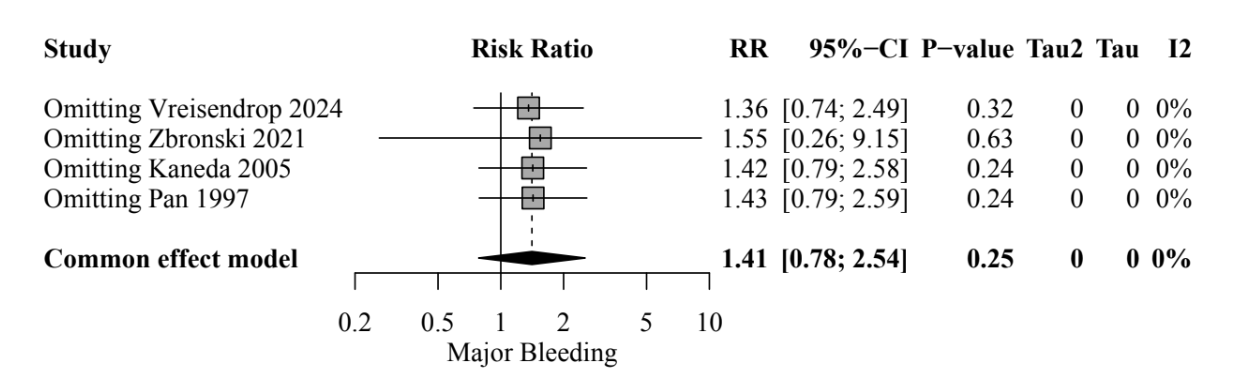


**Figure S9**: Leave-one-out sensitivity analysis of hematoma and pseudoaneurysm.


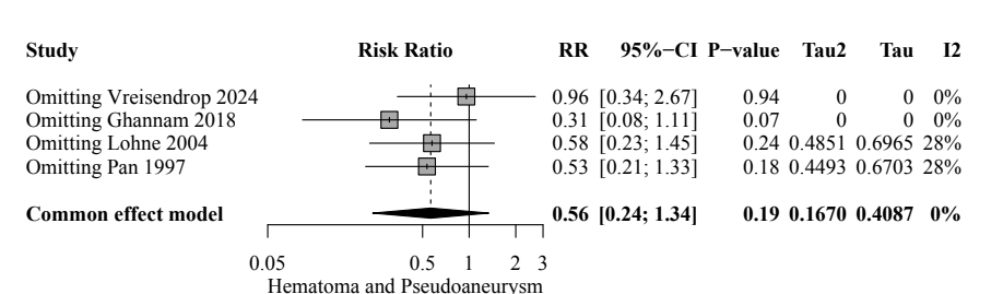


**Figure S10:** Leave-one-out sensitivity analysis of Minor bleeding.

**
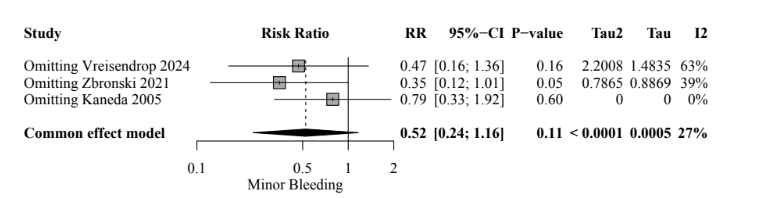
**

**Figure S11:** Leave-one-out sensitivity analysis of All-cause mortality.

**
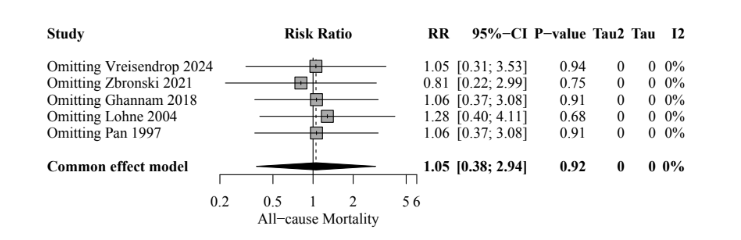
**

**Figure S12:** Leave-one-out sensitivity analysis of Stroke.

**
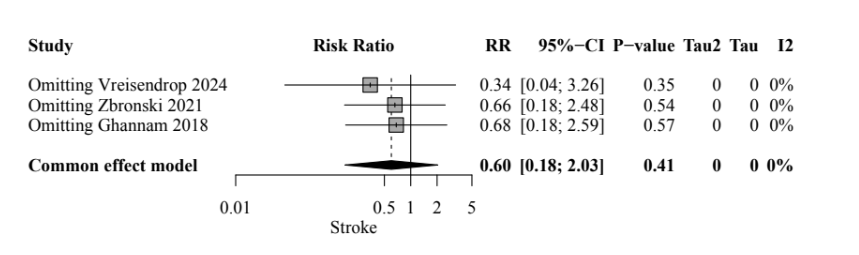
**

**Figure S13:** Leave-one-out sensitivity analysis for length of hospital stay.

**
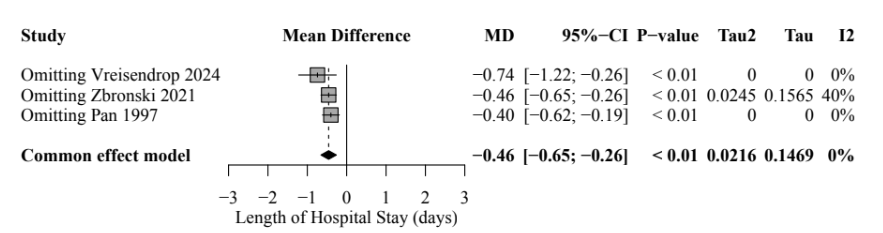
**

**Figure S14:** Subgroup analysis of Major bleeding.


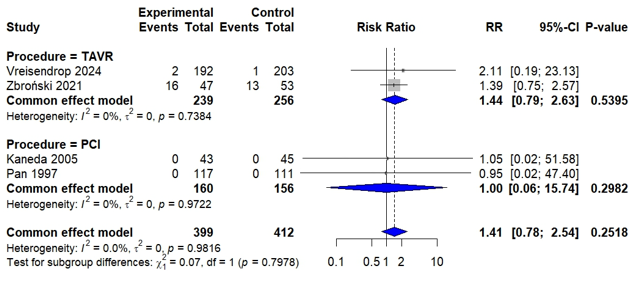


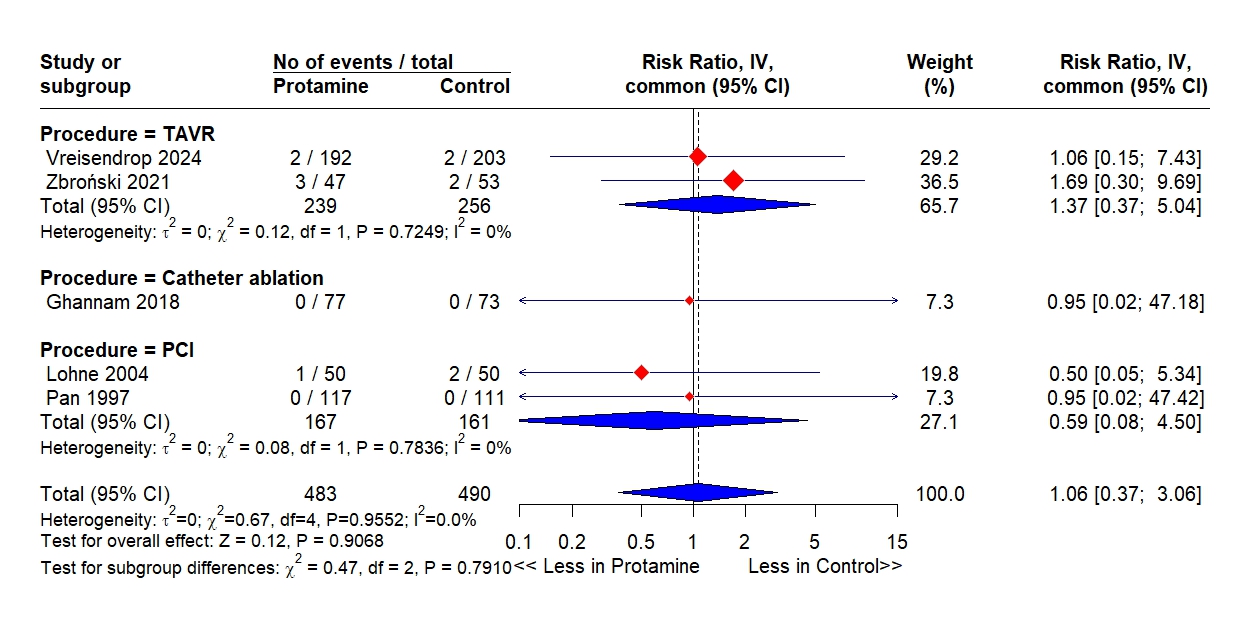
**Figure S15**: Subgroup analysis of All-cause mortality.

**Figure S16:** Subgroup analysis of major vascular complications.

**
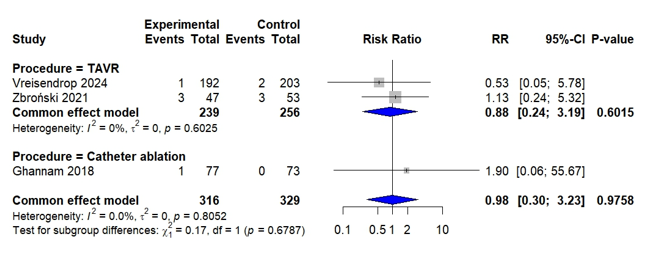
**

**Figure S17:** Subgroup analysis of hematoma and pseudoaneurysm formation.


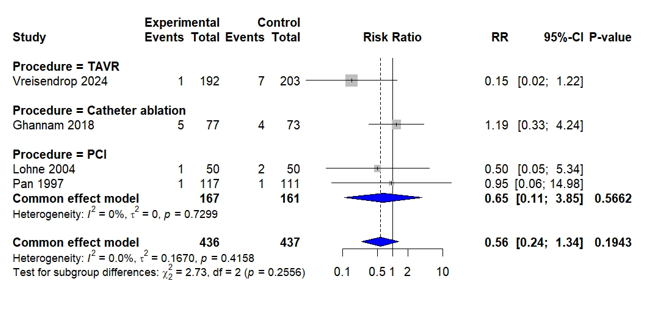


**Figure S18:** Subgroup analysis of minor bleeding.


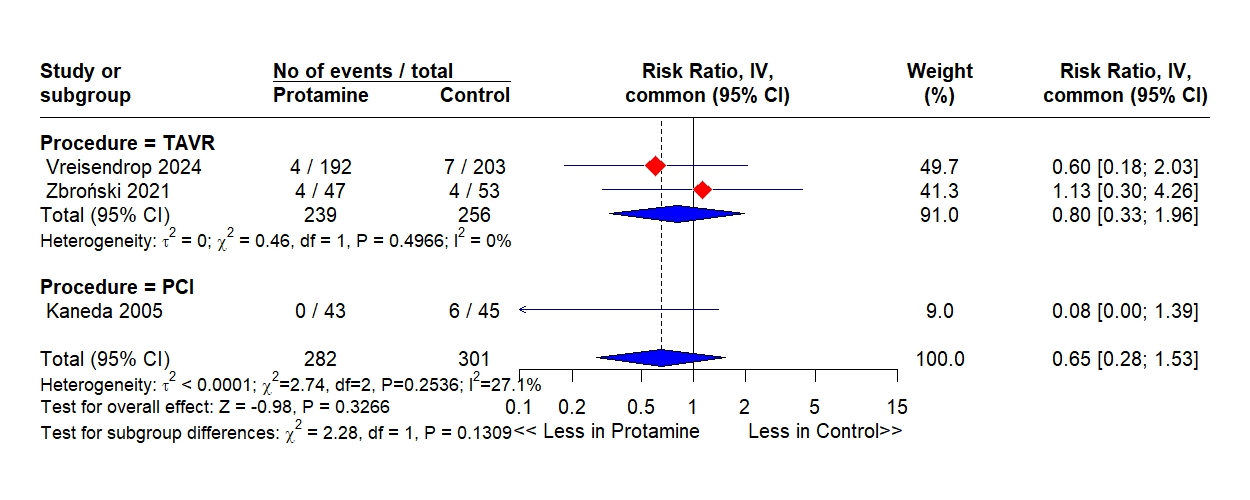


**Figure S19:** Subgroup analysis of stroke.


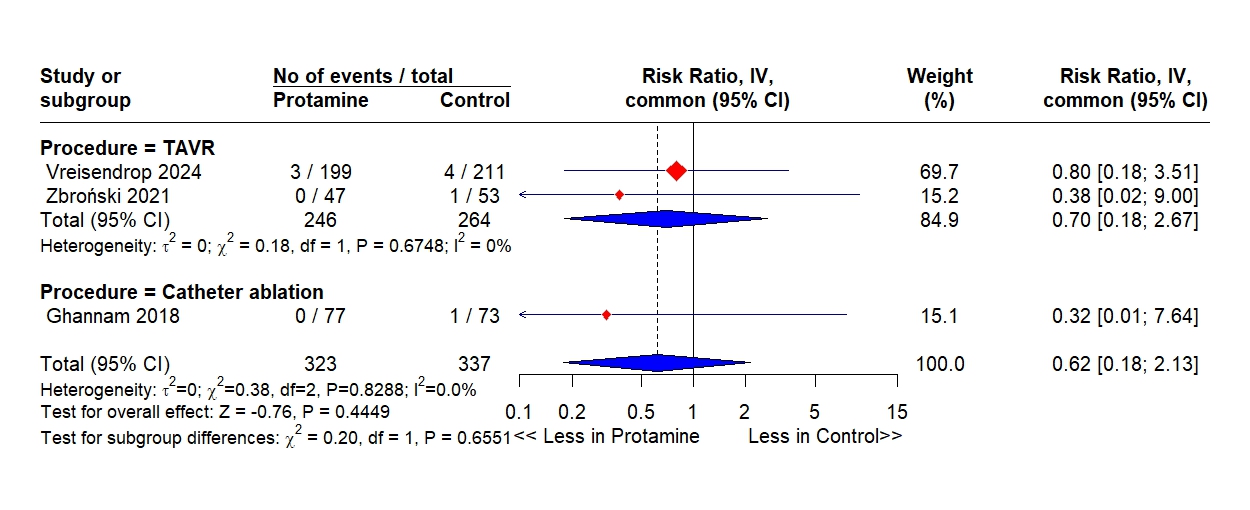


**Figure S20**: Subgroup analysis of Length of hospital.


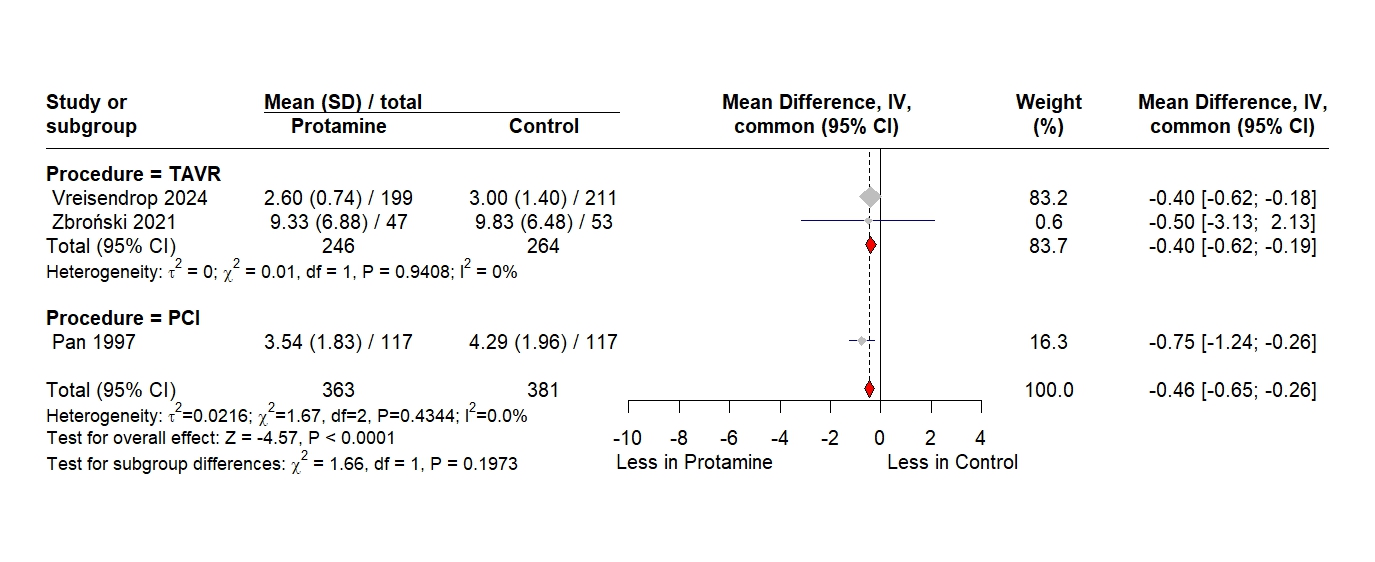

Supplement: Supplementary file 1 — (DOCX 2.23 MB) [file 210_2025_4369_MOESM1_ESM.docx]
